# Anti-Tumorigenic Activity of Chrysin from *Oroxylum indicum* via Non-Genotoxic p53 Activation through the ATM-Chk2 Pathway

**DOI:** 10.3390/molecules23061394

**Published:** 2018-06-08

**Authors:** Mai Nagasaka, Ryoko Hashimoto, Yasumichi Inoue, Kan’ichiro Ishiuchi, Michiyo Matsuno, Yuka Itoh, Muneshige Tokugawa, Nobumichi Ohoka, Daisuke Morishita, Hajime Mizukami, Toshiaki Makino, Hidetoshi Hayashi

**Affiliations:** 1 Department of Cell Signaling, Graduate School of Pharmaceutical Sciences, Nagoya City University, Nagoya 467-8603, Japan; mercredi.le.mai@gmail.com (M.N.); yr.12hh@gmail.com (R.H.); yukait@yamanashi.ac.jp (Y.I.); shogun.fob@me.com (M.T.); daisuke.b.m.1215@gmail.com (D.M.); 2 Department of Innovative Therapeutic Sciences, Cooperative Major in Nanopharmaceutical Sciences, Graduate School of Pharmaceutical Sciences, Nagoya City University, Nagoya 467-8603, Japan; 3 Department of Pharmacognosy, Graduate School of Pharmaceutical Sciences, Nagoya City University; Nagoya 467-8603, Japan; ishiuchi@phar.nagoya-cu.ac.jp (K.I.); makino@phar.nagoya-cu.ac.jp (T.M.); 4 The Kochi Prefectural Makino Botanical Garden, Kochi 781-8125, Japan; matsuno@makino.or.jp (M.M.); hajimem@makino.or.jp (H.M.); 5 Division of Molecular Target and Gene Therapy Products, National Institute of Health Sciences, Kawasaki 210-9501, Japan; n-ohoka@nihs.go.jp

**Keywords:** *Oroxylum indicum*, chrysin, p53, ATM, Chk2, flavonoid

## Abstract

The p53 tumor suppressor plays critical roles in cell cycle regulation and apoptotic cell death in response to various cellular stresses, thereby preventing cancer development. Therefore, the activation of p53 through small molecules is an attractive therapeutic strategy for the treatment of cancers retaining wild-type p53. We used a library of 700 Myanmar wild plant extracts to identify small molecules that induce p53 transcriptional activity. A cell-based screening method with a p53-responsive luciferase-reporter assay system revealed that an ethanol extract of *Oroxylum indicum* bark increased p53 transcriptional activity. Chrysin was isolated and identified as the active ingredient in the *O. indicum* bark extract. A treatment with chrysin increased p53 protein expression and the p53-mediated expression of downstream target genes, and decreased cell viability in MCF7 cells, but not in p53-knockdown MCF7 cells. We also found that chrysin activated the ATM-Chk2 pathway in the absence of DNA damage. Hence, the inactivation of the ATM-Chk2 pathway suppressed p53 activation induced by chrysin. These results suggest the potential of chrysin as an anti-cancer drug through the activation of p53 without DNA damage.

## 1. Introduction

The p53 protein, the guardian of the genome, plays an important role in regulating cell proliferation during various stimuli, including genotoxic stress and oncogenic activation [1,2,3]. It functions as a transcription factor that activates the various genes responsible for cell cycle arrest, senescence, or apoptosis, thereby preventing tumor cell progression [3,4]. *TP53* is mutated in ~50% of all human cancers. However, the incidence of *TP53* mutations differs significantly between cancer types, ranging from nearly universal mutations in serous ovarian cancer to rarely occurring in thyroid cancer [5]. In a large proportion of cancers that retain wild-type (WT) p53, the function of p53 may be compromised by several mechanisms; this offers an attractive strategy for cancer therapy based on p53 activation [6,7]. For example, small-molecule drugs that inhibit the activity of Mdm2, the ubiquitin ligase regulating p53 protein levels, have been developed and entered preclinical trials [8]. Therefore, the development of therapeutic interventions to overcome the inactivation of p53 may lead to the prevention and treatment of cancer.

Phytochemicals are secondary plant metabolites and include flavonoids, triterpenoids, phenols, alkaloids, catechols, saponins, and tannins. Phytochemicals have been widely used for many decades in the prevention and treatment of various ailments, and current evidence suggests the use of phytochemicals as an effective treatment for cancer [9]. Phytochemicals, such as vincristine, taxanes, and camptothecin, which exhibit cytotoxic activities, contribute to the effective treatment of cancer. Therefore, we attempted to identify phytochemicals that induce p53 transcriptional activity from plants. Small molecule activators of p53 that do not cause DNA damage are desired because DNA-damaging p53 activators may increase the risk of developing a second cancer as well as the emergence of drug resistance mutations.

We herein demonstrated that an ethanol extract of *Oroxylum indicum* bark increased p53 transcriptional activity in a screening assay using MCF7 human breast cancer cells with a luciferase-expressing p53-dependent reporter. We isolated active compounds from a methanol extract of *O. indicum* bark through bioassay-guided fractionation. Mass spectrometry (MS) and nuclear magnetic resonance (NMR) analyses revealed that the active compound responsible for p53 activation was 5,7-dihydroxyflavone (chrysin). Chrysin increased p53 protein expression and the p53-mediated expression of downstream target genes, and decreased cell viability in MCF7 cells, but not in p53-knockdown MCF7 cells. Mechanistically, chrysin activated the ATM-Chk2 pathway in the absence of DNA damage. The inactivation of the ATM-Chk2 pathway suppressed chrysin-induced p53 activation. Our results suggest the potential of chrysin as an anti-cancer drug through the activation of p53 without DNA damage.

## 2. Results

### 2.1. Ethanol Extract of O. indicum Bark Increased p53 Transcriptional Activity

**Figure 1:**
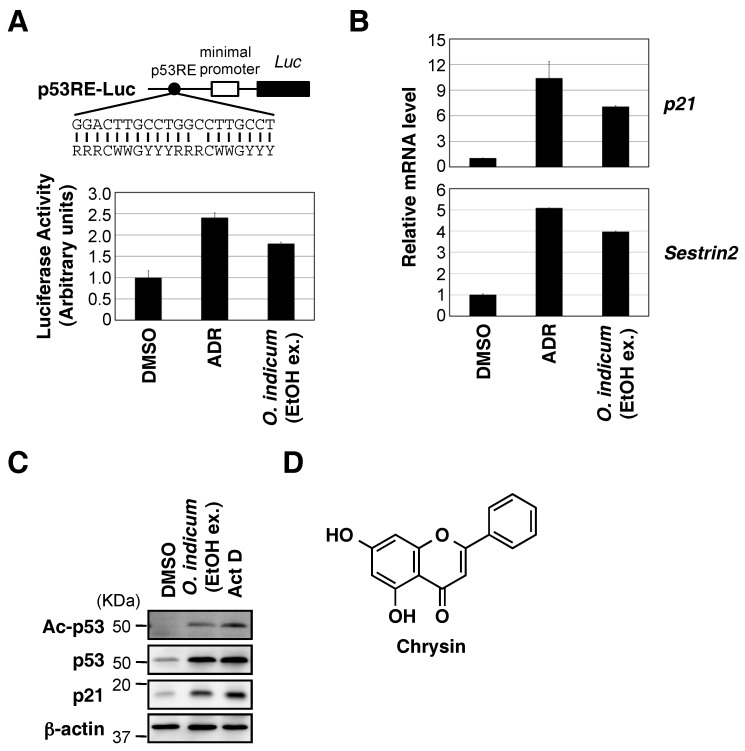
Ethanol extract of *Oroxylum indicum* bark increased p53 transcriptional activity. (**A**) The sequence of p53-responsive element (p53RE) in reporter construct is shown, and the consensus p53 binding sequence (W can be A or T, and R and Y strand for purine and pyrimidine bases, respectively) is shown below. MCF7 cells, stably expressing the p53-responsive luciferase reporter, were treated with ADR (0.3 μM) or the ethanol extract (ex.) of *O. indicum* bark (100 μg/mL). After 8 h, luciferase activities in cell lysates were measured. The experiment was run in triplicate, and data are represented as the mean fold activation ± S.D. (**B**) MCF7 cells were treated with ADR (0.3 μM) or the ethanol extract of *O. indicum* bark (100 μg/mL) for 8 h. The expression of each gene was assessed by qPCR. (**C**) MCF7 cells were treated with the ethanol extract of *O. indicum* bark (100 μg/mL) or actinomycin D (Act D) (10 nM) for 8 h. Cell lysates were immunoblotted with the indicated antibodies. (**D**) Structure of chrysin (5,7-dihydroxyflavone).

To identify small molecules that enhance p53 transcriptional activity, we created a MCF7 cell line that stably expresses a p53-responsive luciferase reporter. This cell line was validated by demonstrating that luciferase activity was induced by the known p53 activator adriamycin (ADR) (Figure 1A). We then screened a library consisting of 700 Myanmar wild plant extracts. We found several extracts that induce p53 activation, many of which involved DNA damage (data not shown). As we discuss below, only the ethanol extract of *O. indicum* bark activated p53 without DNA damage. The ethanol extract of *O. indicum* bark increased p53 transcriptional activity in MCF7 cells (Figure 1A). As shown in Figure 1B,C, a treatment with the *O. indicum* bark extract up-regulated the expression of *p21* and *Sesn2* mRNA, which are well characterized p53 target genes, as well as the p21 protein. This extract also stabilized the p53 protein and increased acetylated p53 levels. Therefore, we focused on the identification of compounds that activate p53 in the ethanol extract of *O. indicum* bark.

To isolate active compounds from *O. indicum* bark, 100 g of *O. indicum* bark was extracted with methanol. The activation of p53 was observed by a treatment with the methanol extract, similar to the ethanol extract (data not shown). The methanol extract was then used as the starting material for activity-guided fractionation. The results of the activity-guided fractionation of the methanol extract and the isolation of constituents are summarized in Figure S1. One hexane fraction (Fraction 13) and four ethyl acetate fractions (Fractions 6–9) had the ability to activate p53. MS and NMR analyses revealed that the active compound responsible for p53 activation was chrysin (Figure 1D).

### 2.2. Chrysin Increased p53 Protein Expression and the p53-Mediated Expression of Downstream Target Genes, and Decreased Cell Viability in a p53-Dependent Manner

**Figure 2:**
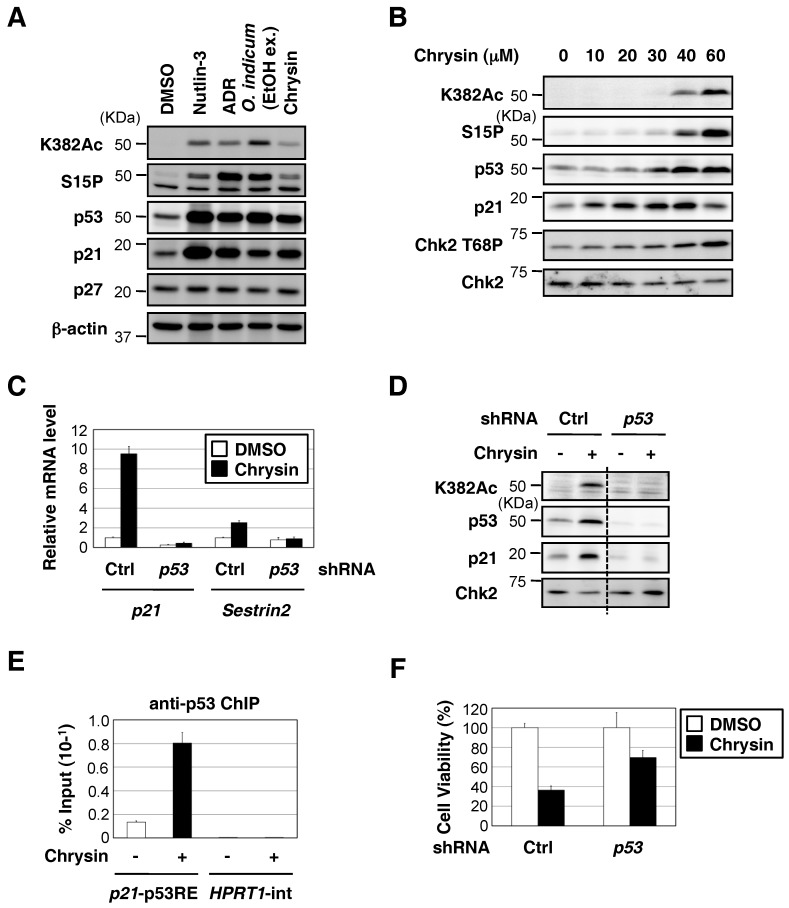
Chrysin increased p53 protein expression and the p53-mediated expression of downstream target genes, and decreased cell viability in a p53-depemdent manner. (**A**) MCF7 cells were treated with Nutlin-3 (10 μM), ADR (0.3 μM), the ethanol extract of *O. indicum* bark (100 μg/mL), or chrysin (40 μM) for 8 h. Cell lysates were immunoblotted with the indicated antibodies. (**B**) MCF7 cells were treated with the indicated doses of chrysin for 8 h. Cell lysates were immunoblotted with the indicated antibodies. (**C**) shcontrol-MCF7 and shp53-MCF7 cells were exposed to 40 μM of chrysin for 8 h. The expression of each gene was assessed by qPCR. (**D**) shcontrol-MCF7 and shp53-MCF7 cells were exposed to 40 μM of chrysin for 8 h. Cell lysates were immunoblotted with the indicated antibodies. (**E**) MCF7 cells were exposed to 40 μM of chrysin for 6 h. ChIP was performed using an anti-p53 antibody, and qPCR was conducted for the indicated promoters. (**F**) shcontrol-MCF7 and shp53-MCF7 cells were exposed to 40 μM of chrysin for 48 h. Cell viability was measured by the WST-8 cell proliferation assay.

**Figure 3:**
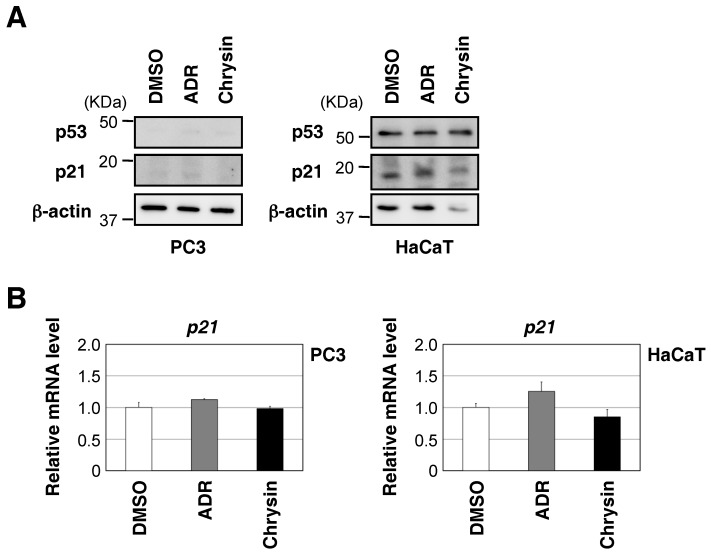
Chrysin did not increase p21 mRNA or p21 protein levels in p53-null and p53-mutant cells. (**A**) PC3 cells and HaCaT cells were treated with ADR (0.3 μM) or chrysin (40 μM) for 8 h. Cell lysates were immunoblotted with the indicated antibodies. (**B**) PC3 cells and HaCaT cells were treated with ADR (0.3 μM) or chrysin (40 μM) for 8 h. The expression of each gene was assessed by qPCR.

Chrysin appears to possess various pharmacological properties, such as antioxidant, immunomodulatory, anticarcinogenic, and proapoptotic activities [10]. We investigated whether chrysin activates p53 in MCF7 cells. Nutlin-3 has been shown to interact with Mdm2, block the Mdm2-p53 interaction, and activate WT p53 without DNA damage [11]. As shown in Figure 2A, the chrysin treatment increased the expression of p53 and p21, similar to the Nutlin-3 treatment, in MCF7 cells. On the other hand, chrysin hardly affected the protein levels of another cyclin-dependent kinase inhibitor, p27. Chrysin also induced the activation of p53 in a dose-dependent manner (Figure 2B). To examine whether the induction of p21 by chrysin was dependent on p53, we knocked down p53 in MCF7 cells. As shown in Figure 2C,D, the knockdown of p53 suppressed the chrysin-induced up-regulation of p21. Since these responses were not observed in p53-null PC-3 cells or p53-mutant HaCaT cells (Figure 3A,B), they were considered to be dependent on p53. Moreover, the chrysin treatment increased the ability of p53 to bind to the p21 promoter, but not to a control region (HPRT1 intron) (Figure 2E). Chrysin decreased cell viability in MCF7 cells (Figure 2F). On the other hand, the knockdown of p53 attenuated the decrease in cell viability caused by the chrysin treatment. These results indicate that chrysin is a WT p53-activating compound and induces cytotoxicity through a p53-dependent pathway.

### 2.3. Biological Evaluation of p53 Activation by Flavonoids in MCF7 Cells

**Figure 4:**
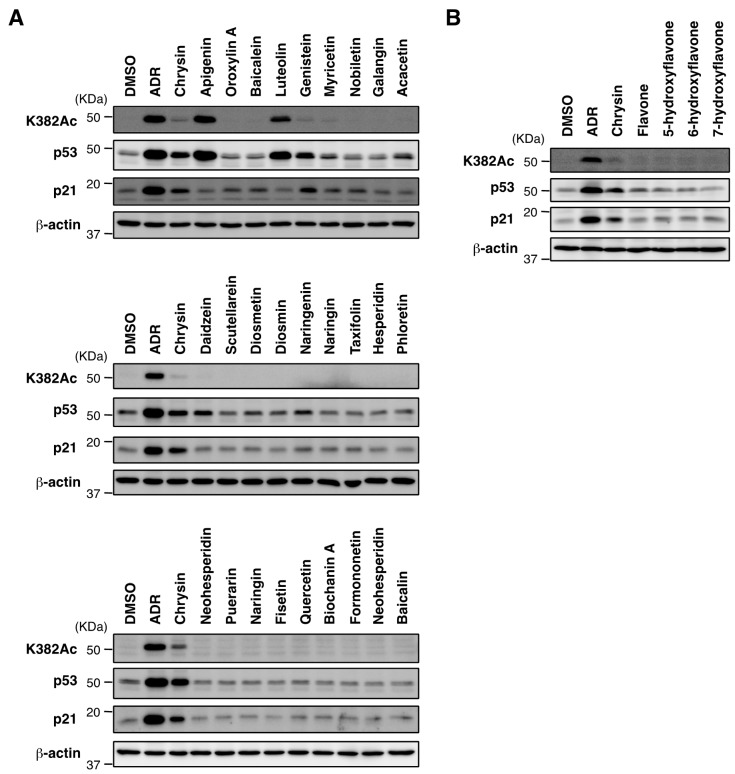
Biological evaluation of p53 activation by flavonoids in MCF7 cells. (**A**) MCF7 cells were treated with ADR (0.3 μM), chrysin (40 μM), or 26 flavonoids (40 μM) for 8 h. Cell lysates were immunoblotted with the indicated antibodies. (**B**) MCF7 cells were treated with ADR (0.3 μM), chrysin (40 μM), or 4 flavones (40 μM) for 8 h. Cell lysates were immunoblotted with the indicated antibodies.

We then investigated whether flavonoids other than chrysin can activate p53 (Figure 4A, Figures S2 and S3). The treatment of apigenin, with a 4′-hydroxy substitution in the B ring of chrysin, or luteolin, with a 3′,4′-dihydroxy substitution in the B ring of chrysin, strongly induced the stabilization and acetylation of p53, but did not induce p21 expression. The treatment of isoflavone genistein caused the stabilization of the p53 protein and induction of p21 in the same manner as the chrysin treatment. However, as described below, genistein induced DNA damage, unlike chrysin. We also tested other flavonoids with various substitution patterns such as hydroxylation, *O*-methylation, *O*-glycosylation, or *C*-glycosylation (Figures S2 and S3), however none inducing the activity of p53 was found (Figure 4A). Especially the results of chrysin derivatives suggest that the ability to activate p53 disappears when substituents are additionally introduced into chrysin. We next investigated whether flavonoids with fewer hydroxy groups than chrysin can activate p53 (Figures S2 and S3). As shown in Figure 4B, flavone, 5-hydroxyflavone, 6-hydroxyflavone, and 7-hydroxyflavone did not activate p53, which indicated that 5,7-dihydroxy groups of chrysin play an important role in the activation ability of p53. Collectively, these results suggest that chrysin is a compound among flavonoids that selectively activates the p53 pathway.

### 2.4. Chrysin Activated the ATM-Chk2 Pathway in the Absence of DNA Damage

**Figure 5:**
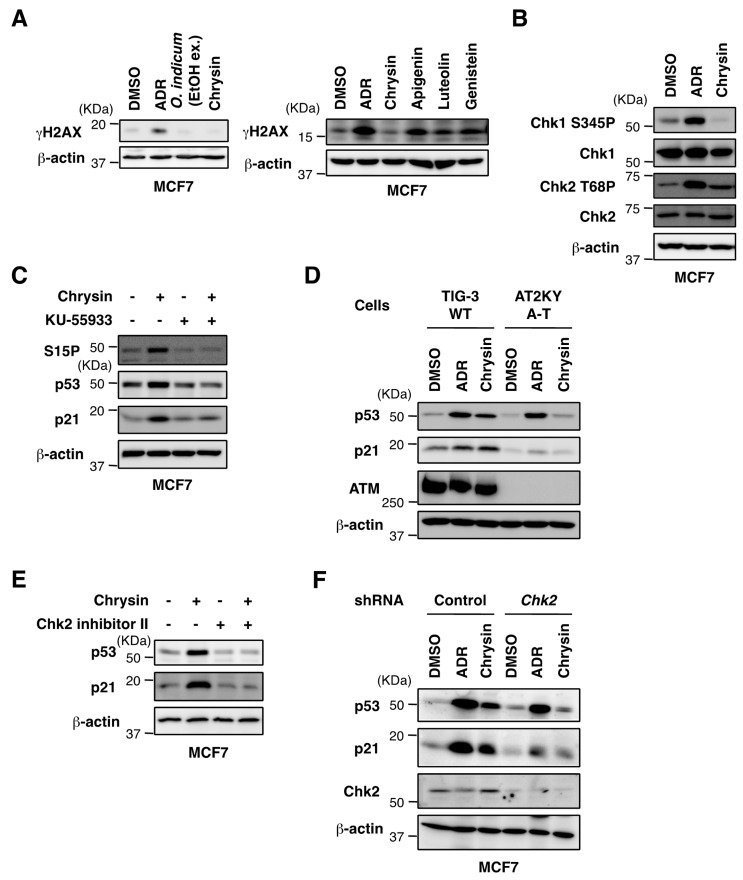
Chrysin activated the ATM-Chk2 pathway in the absence of DNA damage. (**A**) MCF7 cells were treated with ADR (0.3 μM), the ethanol extract of *O. indicum* bark (100 μg/mL), chrysin (40 μM), or flavonoids (40 μM) for 8 h. Cell lysates were immunoblotted with the indicated antibodies. (**B**) MCF7 cells were treated with ADR (0.3 μM) or chrysin (40 μM) for 8 h. Cell lysates were immunoblotted with the indicated antibodies. (**C**) MCF7 cells were pretreated with KU-55933 (10 μM) for 1 h and subsequently treated with chrysin (40 μM) for 8 h. Cell lysates were immunoblotted with the indicated antibodies. (**D**) TIG-3 cells and AT2KY cells were treated with ADR (0.3 μM) or chrysin (40 μM) for 8 h. Cell lysates were immunoblotted with the indicated antibodies. (**E**) MCF7 cells were pretreated with Chk2 inhibitor II (10 μM) for 1 h and subsequently treated with chrysin (40 μM) for 8 h. Cell lysates were immunoblotted with the indicated antibodies. (**F**) shcontrol-MCF7 and shChk2-MCF7 cells were treated with ADR (0.3 μM) or chrysin (40 μM) for 8 h. Cell lysates were immunoblotted with the indicated antibodies.

We noted that the ethanol extract of *O. indicum* bark or chrysin induced the phosphorylation of p53 at Ser15 (Figure 2A,B). Ser15 phosphorylation has been shown to stimulate the association of p53 with histone acetyltransferases p300 and CBP and to regulate the transactivation properties of p53 [12,13]. Ser15 is the primary target of the DNA damage response on the p53 protein and is phosphorylated by ATM and ATR protein kinases [14]. On the other hand, some phytochemicals (e.g., resveratrol, genistein, and baicalein) are known to be potent DNA-damaging agents [15]. Therefore, we investigated whether chrysin causes DNA damage. The treatment with ADR, apigenin, luteolin or genistein resulted in the significant induction of the double-stranded DNA break marker, H2AX phosphorylation (γH2AX) (Figure 5A). However, the treatment with chrysin did not increase phosphorylated H2AX at a concentration of 40 μM. Chrysin induced the phosphorylation of Chk2, but not Chk1 (Figure 2B and Figure 5B).

The ATM-Chk2 and ATR-Chk1 pathways are activated by DNA double-strand breaks and single-strand DNA, respectively [16]. We investigated whether ATM is required for the phosphorylation of p53 at Ser15 by chrysin. As shown in Figure 5C, KU-55933, a specific ATM inhibitor [17], blocked the Ser15 phosphorylation of p53 by chrysin. KU-55933 also suppressed the chrysin-induced stabilization of the p53 protein and p21 induction. In addition, the stabilization of the p53 protein and p21 induction were observed in human diploid fibroblasts, TIG-3 cells, but not in A-T fibroblasts, AT2KY cells, subjected to the chrysin treatment (Figure 5D). These results suggest that ATM plays an important role in the activation of p53 by chrysin.

Since Chk2 serves as an ATM downstream effector to mediate the activation of p53, we investigated whether Chk2 is responsible for the activation of p53 by chrysin. As shown in Figure 5E, Chk2 inhibitor II [18] suppressed the chrysin-induced stabilization of the p53 protein and p21 induction. Moreover, the stabilization of the p53 protein and induction of p21 by the chrysin treatment were weakened in Chk2-depleted MCF7 cells (Figure 5F). Collectively, these results prompted us to conclude that chrysin activates the ATM-Chk2 pathway and induces p53 activation without DNA damage.

## 3. Discussion

The tumor suppressor p53 functions primarily as a transcription factor. p53 activates the various genes responsible for cell cycle arrest, senescence, or apoptosis to prevent tumor cell progression [3,4]. Among them, the cyclin-dependent kinase (CDK) inhibitor p21 plays an important role in the regulation of the cell cycle. The activation of the G1 cyclin/CDK complex is necessary for progression from the G1 phase to the S phase, whereas p21 binds to these cyclin/CDK complexes and suppresses CDK activity, leading to G1 arrest [19]. In the present study, we showed that chrysin obtained from the ethanol extract of *O. indicum* bark increased p53 transcriptional activity and induced the up-regulation of p21 by enhancing p53 binding to the *p21* promoter (Figure 2). Since these responses were not observed in *p53*-null PC-3 cells or *p53*-mutant HaCaT cells, they were considered to be p53-dependent. Thus, chrysin is a WT p53-activating compound that induces cytotoxicity through a p53-dependent pathway.

The cell has a monitoring mechanism called the “DNA damage checkpoint response” that detects DNA damage caused by various stresses and stops the cell cycle until damage has recovered [20]. When ATM or ATR is activated in response to DNA damage, they phosphorylate H2AX around the DNA damage site [14]. This reaction triggers the activation of various checkpoint kinases, including ATM, and downstream signal transmission. Ser15 is the primary target of the DNA damage response on the p53 protein and is phosphorylated by ATM and ATR protein kinases [14]. Furthermore, ATM is required for the phosphorylation of Ser20 through the activation of Chk2. As a result, activated p53 induces p21 and stops the cell cycle, and induces apoptosis in the case of severe damage. We showed that chrysin enhanced Chk2 phosphorylation, whereas the phosphorylation of H2AX did not occur. To investigate whether ATM is required for the phosphorylation of p53 at Ser15 by chrysin, we conducted experiments using the ATM inhibitor KU-55933 and cells derived from A-T patients lacking ATM. As shown in Figure 5C,D, ATM is necessary for the activation of p53 and induction of p21 by chrysin. We also found that Chk2 is essential for the activation of p53 and induction of p21 by chrysin using a technique with a Chk2 kinase inhibitor and the knockdown of Chk2 (Figure 5E,F). These results indicate that chrysin is a compound that induces p53 activation without DNA damage. It has been demonstrated that ATM can also be activated by a few non-canonical inducers, such as oxidative stress and hypoxia [21,22]. The mechanism of activation of ATM without DNA damage by chrysin need to be examined in detail in future.

Chrysin is a dietary phytochemical that is abundantly present in many natural products, including propolis, blue passion flower (*Passiflora caerulea*), and honey, which have great economic value and medicinal impact [10]. Chrysin has many pharmacological properties, such as antioxidant, anti-inflammatory, and anticancer activities [23]. Previous studies reported that chrysin exerted cytotoxic effects on human colon cancer in vitro [24,25]. The anti-cancer potential of chrysin has also been demonstrated in several cell lines [26]. Chrysin has been suggested to regulate several intracellular signal transduction pathways, such as the MAPK pathway and NF-κB [10]. The present results indicate that chrysin inhibits cell proliferation via the activation of p53. Several studies suggesting that chrysin activates p53 have also been reported. Zhang et al. have shown that chrysin induces apoptosis in hepatocellular carcinoma cells by regulating the p53/Bcl-2/caspase-9 signaling pathway, but the underlying molecular mechanisms have not been clarified [27]. Another study showed that chrysin enhances p53 expression in ATL cells via inhibition of Mdm2 expression [28]. Li et al. showed that the combination of chrysin and cisplatin promotes the apoptosis of hepatocellular carcinoma HepG2 cells by upregulating p53 [29]. Thus, chrysin has potential as an anti-cancer drug through its activation of p53. Some foods that abundantly include chrysin may be useful to prevent the development of cancer. However, due to its limited bioavailability and absorption, few studies have investigated the therapeutic value of chrysin [10]. To overcome poor bioavailability, drug delivery systems for chrysin (e.g., liposomes, micelles, and nanoparticles as carriers) have been extensively evaluated [30,31,32]. Therefore, the bioavailability and therapeutic efficacy of chrysin may be improved by a nanoformulation even at lower doses [10].

In conclusion, chrysin increased p53 protein expression and the p53-mediated expression of downstream target genes, and decreased cell viability in MCF7 cells. Chrysin activates the ATM-Chk2 pathway and induces p53 activation without DNA damage. Given its cytostatic effects on cancer cells expressing WT p53, the characterization of the effects of chrysin will provide insights into novel pathways regulating the p53 tumor suppressor. Further studies are needed to establish the precise mechanisms of action of chrysin on the activation of the ATM-Chk2 pathway.

## 4. Materials and Methods

### 4.1. Cell Lines, Plasmids, and RNA Interference

MCF7, HaCaT, TIG-3, and AT2KY cells were maintained in Dulbecco’s modified Eagle’s medium (Nacalai Tesque, Kyoto, Japan) supplemented with 4.5 g/L glucose, 10% fetal bovine serum (FBS) (Sigma, St. Louis, MO, USA), 100 U/mL of penicillin G, and 100 μg/mL of streptomycin as previously described [33,34]. PC3 cells were cultured in Roswell Park Memorial Institute 1640 medium (Nacalai Tesque) containing 10% FBS and penicillin/streptomycin. Cells were grown in a 5% CO_2_ atmosphere at 37 °C.

To construct a lentiviral expression vector containing the p53-responsive reporter luciferase, cDNA encoding p53RE-Luc2 [35] was inserted into the ClaI/XbaI of pLenti6/V5-DEST (Invitrogen, Carlsbad, CA, USA). This sequence is from a putative replication origin of the human ribosomal gene cluster, selected for p53 binding [36]. MCF7 cells were infected with viral particles according to standard protocols and selected with 8 μg/mL blasticidin.

Regarding the stable knockdown in MCF7 cells, shRNA against *p53* (5′-gatccccGACTCCAGTGGTAATCTACttcaagagaGTAGATTACCACTGGAGTCtttttggaaa-3′) [37] was expressed in the retroviral vector pSUPERretro (Oligoengine, Seattle, WA, USA). The sequence provided is the primer sequence cloned into the pSUPERretro vector, with the uppercase letters representing sequences complementary to the target gene. MCF7 cells were infected with viral particles and selected with 0.5 μg/mL puromycin. shcontrol-MCF7 and shChk2-MCF7 cells were previously established and reported [33].

### 4.2. RNA Extraction, Reverse Transcription, and Quantitative PCR (qPCR)

Total RNA extraction was performed as previously described [38]. First-strand cDNA was synthesized with the PrimeScript first-strand cDNA Synthesis Kit (TaKaRa Bio Inc., Shiga, Japan) as previously described [35]. qPCR was performed as previously described [39]. The following primer sequences were used: human *p21*, 5′-GATTTCTACCACTCCAAACGCC-3′ (forward) and 5′-AGAAGATGTAGAGCGGGC-3′ (reverse) [40]; human *Sestrin2*, 5′-GACCATGGCTACTCGCTGAT-3′ (forward) and 5′-GCTGCCTGGAACTTCTCATC-3′ (reverse) [41]; human *HPRT1*, 5′-TTTGCTTTCCTTGGTCAGGC-3′ (forward) and 5′-GCTTGCGACCTTGACCATCT-3′ (reverse) [40]. The specificities of the detected signals were confirmed by a dissociation curve, which consisted of a single peak. Values were normalized by *HPRT1*.

### 4.3. Immunochemical Methods and Antibodies

Immunoblotting was performed as previously described [42]. The following commercially available antibodies were used: anti-p21 (C-19; Santa Cruz Biotechnology, Santa Cruz, CA, USA), horseradish peroxidase-conjugated anti-p53 antibody (DO-1; Santa Cruz Biotechnology), anti-p53 (DO-1; EMD Millipore, Darmstadt, Germany), anti-p27 (Clone 57/Kip1/p27; BD Bioscience, Franklin Lakes, NJ, USA), anti-acetyl-p53 (Lys382) (#2525; Cell Signaling Technology, Beverly, MA, USA), anti-phospho-p53 (Ser15) (#9284; Cell Signaling Technology), anti-phospho-Chk2 (Thr68) (#2661; Cell Signaling Technology), anti-Chk2 (#2662; Cell Signaling Technology), anti-phospho-Chk1 (Ser345) (#2348; Cell Signaling Technology), anti-Chk1 (#2360; Cell Signaling Technology), anti-ATM (#2873; Cell Signaling Technology), anti-phospho-histone H2AX (Ser139) (#9718; Cell Signaling Technology), and anti-β-actin (2F3) (Wako, Osaka, Japan).

### 4.4. Reporter Assay

The luciferase assay was performed as previously described [35].

### 4.5. Chromatin Immunoprecipitation Assay

The chromatin immunoprecipitation assay was performed as previously described [40]. Purified DNA was analyzed by qPCR. The following primer sequences were used: human *p21* promoter (p53RE), 5′-GTGGCTCTGATTGGCTTTCTG-3′ (forward) and 5′-CTGAAAACAGGCAGCCCAAG-3′ (reverse); human *HPRT1* first intron, 5′-TGTTTGGGCTATTTACTAGTTG-3′ (forward) and 5′-ATAAAATGACTTAAGCCCAGAG-3′ (reverse) [40].

### 4.6. Cell Viability Assay

Cell viability was assessed using water-soluble tetrazolium WST-8 according to the manufacturer’s instructions (Dojindo, Kumamoto, Japan). Cells were seeded at a concentration of 5 × 10^3^ cells per well on a 96-well plate. After 24 h, cells were treated with chrysin for 48 h. The WST-8 reagent was added and cells were incubated at 37 °C for 2 h in a humidified atmosphere of 5% CO_2_. The absorbance at 450 nm of the medium was measured. The percentage of cell viability was normalized to vehicle-treated cells [34].

### 4.7. Myanmar Natural Plant Extract Library

The plant materials used to construct the extract library, including *O. indicum* bark, were collected in Myanmar under the Memorandum of Understanding between The Kochi Prefectural Makino Botanical Garden (MBK), Japan and Forestry Department, The Ministry of Natural Resources and Environmental Conservation, Myanmar. Voucher specimens of the plant materials were deposited in the herbarium of MBK. Plant samples were supplied as an ethanol extract to the laboratory.

### 4.8. Plant Material

*O. indicum* (L.) Kurz was collected in the Mandalay Division in Myanmar. Botanical identification was performed by Dr. Nobuyuki Tanaka, MBK.

### 4.9. Spectroscopic Experimental Procedures

NMR spectra were recorded on an Agilent Varian VNS500 spectrometer. Chemical shifts (ppm) were referenced to residual solvent peaks (*δ*_H_ 2.50 and *δ*_C_ 39.5 for DMSO-*d*_6_). Negative-mode ESITOFMS was obtained on a JEOL JMS-T100LP AccuTOF LC-plus 4G spectrometer using a sample dissolved in MeOH.

### 4.10. Extraction and Isolation of Chrysin from O. indicum Bark

The dried bark of *O. indicum* (100 g) was extracted with methanol at room temperature and evaporated in vacuo. The concentrated methanol extract (16.1 g) was successively partitioned among 1-hexane, ethyl acetate, 1-butanol, and H_2_O to give active residues of the 1-hexane and ethyl acetate fractions, and inactive residues of the 1-butanol and H_2_O fractions. The hexane-soluble fraction was separated over a silica gel column using a stepwise gradient of increasing polarity from 100% hexane to 100% ethanol into 15 sub-fractions. The ethyl acetate fraction was separated over a silica gel column using a stepwise gradient of increasing polarity from 100% ethyl acetate to 100% methanol into 13 sub-fractions. One hexane fraction (fraction 13) and four ethyl acetate fractions (fractions 6–9) were discovered to have the ability to activate p53 as assessed by immunoblotting. ESI-MS and NMR analyses revealed that the active compound responsible for p53 activation was chrysin. Chrysin: ^1^H-NMR (DMSO-*d*_6_, 500 MHz) *δ* 12.8 (1H, s), 8.07 (2H, dd 7.0, 1.5 Hz), 7.55–7.63 (3H), 6.98 (1H, s), 6.53 (1H, d 2.0 Hz), 6.22 (1H, d 2.0 Hz); ^13^C-NMR (DMSO-*d*_6_, 125 MHz) *δ* 181.9, 164.5, 163.2, 161.5, 157.5, 132.1, 130.7, 129.2, 126.5, 105.2, 104.0, 99.1, 94.2; ESITOFMS *m/z* 253 (M − H)^−^; HRESITOFMS *m/z* 253.0495 [(M − H)^−^; calcd for C_15_H_9_O_4_, 253.0501].

### 4.11. Chemicals

Chrysin, apigenin, luteolin, genistein, and KU-55933 were purchased from Wako. 6-Hydroxyflavone and 7-hydroxyflabone were purchased from Tokyo Kasei Kogyo (Tokyo, Japan). Flavone was purchased from Nacalai Tesque. Oroxylin A was purchased from AdooQ Bioscience (Irvine, CA, USA). All other chemicals were purchased from Sigma.

## Supplementary Materials

Figure S1. Activity-guided fractionation of the methanol extract and isolation of active principles. Figure S2. The structure of all flavonoids used in Figure 4. Figure S3. Chemical structure of flavone.

## References

[1] Hainaut P., Hollstein M. (2000). P53 and human cancer: The first ten thousand mutations. Adv. Cancer Res..

[2] Fridman J.S., Lowe S.W. (2003). Control of apoptosis by p53. Oncogene.

[3] Vousden K.H., Prives C. (2009). Blinded by the light: The growing complexity of p53. Cell.

[4] Menedez D., Inga A., Resnick M.A. (2009). The expanding universe of p53 targets. Nat. Rev. Cancer.

[5] Burgess A., Chia K.M., Haupt S., Thomas D., Haupt Y., Lim E. (2016). Clinical overview of MDM2/X-targeted therapies. Front. Oncol..

[6] Toledo F., Wahl G.M. (2006). Regulating the p53 pathway: In vitro hypothesis, in vivo veritas. Nat. Rev. Cancer.

[7] Wade M., Li Y.C., Wahl G.M. (2013). MDM2, MDMX and p53 in oncogenesis and cancer therapy. Nat. Rev. Cancer.

[8] Cheok C.F., Verma C.S., Baselga J., Lane D.P. (2011). Translating p53 into the clinic. Nat. Rev. Clin. Oncol..

[9] Suvarna V., Murahari M., Khan T., Chaubey P., Sangave P. (2017). Phytochemicals and PI3K inhibitors in cancer-an insight. Front. Pharmacol..

[10] Mani R., Natesan V. (2018). Chrysin: Sources, beneficial pharmacological activities, and molecular mechanism of action. Phytochemistry.

[11] Vassilev L.T., Vu B.Y., Graves B., Carvajal D., Podlaski F., Filipovic Z., Kong N., Kammlott U., Lukacs C., Klein C. (2004). In vivo activation of the p53 pathway by small-molecule antagonists of MDM2. Science.

[12] Meek D.W., Anderson C.W. (2009). Posttranslational modification of p53: Cooperative integrators of function. Cold Spring Harb. Perspect. Biol..

[13] Kruse J.P., Gu W. (2009). Modes of p53 regulation. Cell.

[14] Blackford A.N., Jackson S.P. (2017). ATM, ATR, and DNA-PK: The trinity at the heart of the DNA damage response. Mol. Cell.

[15] Fox J.T., Sakamuru S., Huang R., Teneva N., Simmons S.O., Xia M., Tice R.R., Austin C.P., Myung K. (2012). High-throughput genotoxicity assay identifies antioxidants as inducers of DNA damage response and cell death. Proc. Natl. Acad. Sci. USA.

[16] Manic G., Obrist F., Sistigu A., Vitale I. (2015). Trial Watch: Targeting ATM-CHK2 and ATR-CHK1 pathways for anticancer therapy. Mol. Cell. Oncol..

[17] Hickson I., Zhao Y., Richardson C.J., Green S.J., Martin N.M., Orr A.I., Reaper P.M., Jackson S.P., Curtin N.J., Smith G.C. (2004). Identification and characterization of a novel and specific inhibitor of the ataxia-telangiectasia mutated kinase ATM. Cancer Res..

[18] Arienti K.L., Brunmark A., Axe F.U., McClure K., Lee A., Blevitt J., Neff D.K., Huang L., Crawford S., Pandit C.R. (2005). Checkpoint kinase inhibitors: SAR and radioprotective properties of a series of 2-arylbenzimidazoles. J. Med. Chem..

[19] Deng C., Zhang P., Harper J.W., Elledge S.J., Leder P. (1995). Mice lacking p21CIP1/WAF1 undergo normal development, but are defective in G1 checkpoint control. Cell.

[20] Bartek J., Lukas J. (2007). DNA damage checkpoint: From initiation to recovery or adaptation. Curr. Opin. Cell Biol..

[21] Bencokova Z., Kaufmann M.R., Pires I.M., Lecane P.S., Giaccia A.J., Hammond E.M. (2009). ATM activation and signaling under hypoxic conditions. Mol. Cell. Biol..

[22] Guo Z., Kozlov S., Lavin M.F., Person M.D., Paull T.T. (2010). ATM activation by oxidative stress. Science.

[23] Davatgaran-Taghipour Y., Masoomzadeh S., Farzaei M.H., Bahramsoltani R., Karimi-Soureh Z., Rahimi R., Abdollahi M. (2017). Polyphenol nanoformulations for cancer therapy: Experimental evidence and clinical perspective. Int. J. Nanomed..

[24] Khoo B.Y., Chua S.L., Balaram P. (2010). Apoptotic effects of chrysin in human cancer cell lines. Int. J. Mol. Sci..

[25] Bahadori M., Baharara J., Amini E. (2016). Anticancer properties of chrysin on colon cancer cells, in vitro and in vivo with modulation of Caspase-3, -9, Bax and Sall4. Iran. J. Biothechnol..

[26] Yang F., Jin H., Pi J., Jiang J.H., Liu L., Bai H.H., Yang P.H., Cai J.Y. (2013). Anti-tumor activity evaluation of novel chrysin-organogermanium(IV) complex in MCF-7 cells. Bioorg. Med. Chem. Lett..

[27] Zhang Q., Ma S., Liu B., Liu J., Zhu R., Li M. (2016). Chrysin induces cell apoptosis via activation of the p53/Bcl-2/caspase-9 pathway in hepatocellular carcinoma cells. Exp. Ther. Med..

[28] Ding J., Polier G., Köhler R., Giaisi M., Krammer P.H., Li-Weber M. (2012). Wogonin and related natural flavones overcome tumor necrosis factor-related apoptosis-inducing ligand (TRAIL) protein resistance of tumors by down-regulation of c-FLIP protein and up-regulation of TRAIL receptor 2 expression. J. Biol. Chem..

[29] Li X., Huang J.M., Wang J.N., Xiong X.K., Yang X.F., Zou F. (2015). Combination of chrysin and cisplatin promotes the apoptosis of HepG2 cells by up-regulating p53. Chem. Biol. Interact..

[30] Anari E., Akbarzadeh A., Zarghami N. (2016). Chrysin-loaded PLGA-PEG nanoparticles designed for enhanced effect on the breast cancer cell line. Artif. Cells Nanomed. Biotechnol..

[31] Mohammadinejad S., Akbarzadeh A., Rahmati-Yamchi M., Hatam S., Kachalaki S., Zohreh S., Zarghami N. (2015). Preparation and evaluation of chrysin encapsulated in PLGA-PEG nanoparticles in the T47-D breast cancer cell line. Asian Pac. J. Cancer Prev..

[32] Zheng H., Li S., Pu Y., Lai Y., He B., Gu Z. (2014). Nanoparticles generated by PEG-Chrysin conjugates for efficient anticancer drug delivery. Eur. J. Pharm. Biopharm..

[33] Inoue Y., Kitagawa M., Taya Y. (2007). Phosphorylation of pRB at Ser612 by Chk1/2 leads to a complex between pRB and E2F-1 after DNA damage. EMBO J..

[34] Inoue Y., Kawachi C., Ohkubo T., Nagasaka M., Ito S., Fukuura K., Itoh Y., Ohoka N., Morishita D., Hayashi H. (2017). The CDK inhibitor p21 is a novel target gene of ATF4 and contributes to cell survival under ER stress. FEBS Lett..

[35] Kawarada Y., Inoue Y., Kawasaki F., Fukuura K., Sato K., Tanaka T., Itoh Y., Hayashi H. (2016). TGF-beta induces p53/Smads complex formation in the PAI-1 promoter to active transcription. Sci. Rep..

[36] Kern S.E., Kinzler K.W., Bruskin A., Jarosz D., Friedman P., Prives C., Vogelstein B. (1991). Identification of p53 as a sequence-specific DNA-binding protein. Science.

[37] Brummelkamp T.R., Bernards R., Agami R. (2002). A system for stable expression of short interfering RNAs in mammalian cells. Science.

[38] Inoue Y., Abe K., Onozaki K., Hayashi H. (2015). TGF-beta decreases the stability of IL-18-induced IFN-gamma mRNA through the expression of TGF-beta-induced tristetraprolin in KG-1 cells. Biol. Pharm. Bull..

[39] Miyajima C., Inoue Y., Hayashi H. (2015). Pseudokinase tribbles1 (TRB1) negatively regulates tumor-suppressor activity of p53 through p53 deacetylation. Biol. Pharm. Bull..

[40] Inoue Y., Iemura S.I., Natsume T., Miyazawa K., Imamura T. (2011). Suppression of p53 activity through the cooperative action of Ski and histone deacetylase SIRT1. J. Biol. Chem..

[41] Ding B., Parmigiani A., Divakaruni A.S., Archer K., Murphy A.N., Budanov A.V. (2016). Sestrin2 is induced by glucose starvation via the unfolded protein response and protects cells from non-canonical necroptotic cell death. Sci. Rep..

[42] Inoue Y., Itoh Y., Abe K., Okamoto T., Daitoku H., Fukamizu A., Onozaki K., Hayashi H. (2017). Smad3 is acetylated by p300/CBP to regulate its transactivation activity. Oncogene.

