# Supplementary material for: Anti-Tumorigenic Activity of Chrysin from Oroxylum indicum via Non-Genotoxic p53 Activation through the ATM-Chk2 Pathway
Source: Molecules. 2018 Jun 8;23(6):1394. doi: 10.3390/molecules23061394 (PMC6099937; doi:10.3390/molecules23061394)
Supplement: Supplementary file 1 [file molecules-23-01394-s001.pdf]

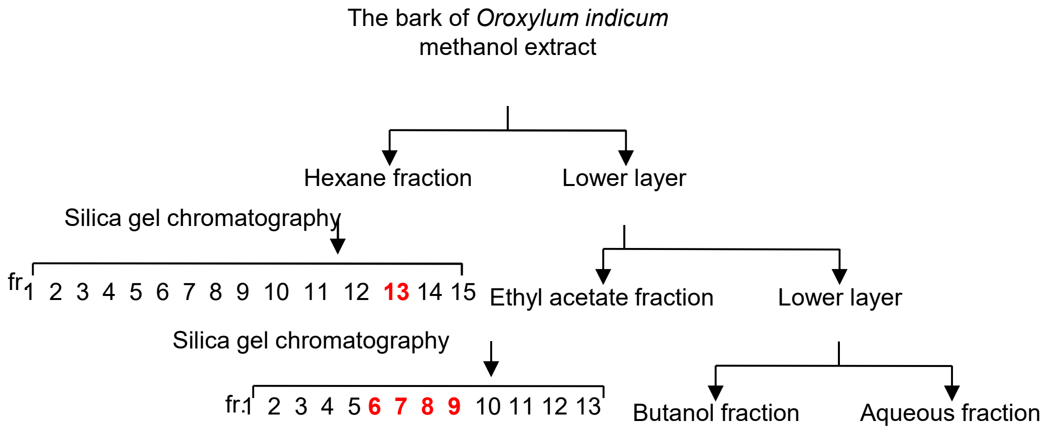

Figure S1. Activity-guided fractionation of the methanol extract and isolation of active principles. One hexane fraction (fraction 13) and four ethyl acetate fractions (fractions 6–9) were discovered to have the ability of p53 activation as assessed by immunoblotting. Fractions containing active ingredients are shown in red.

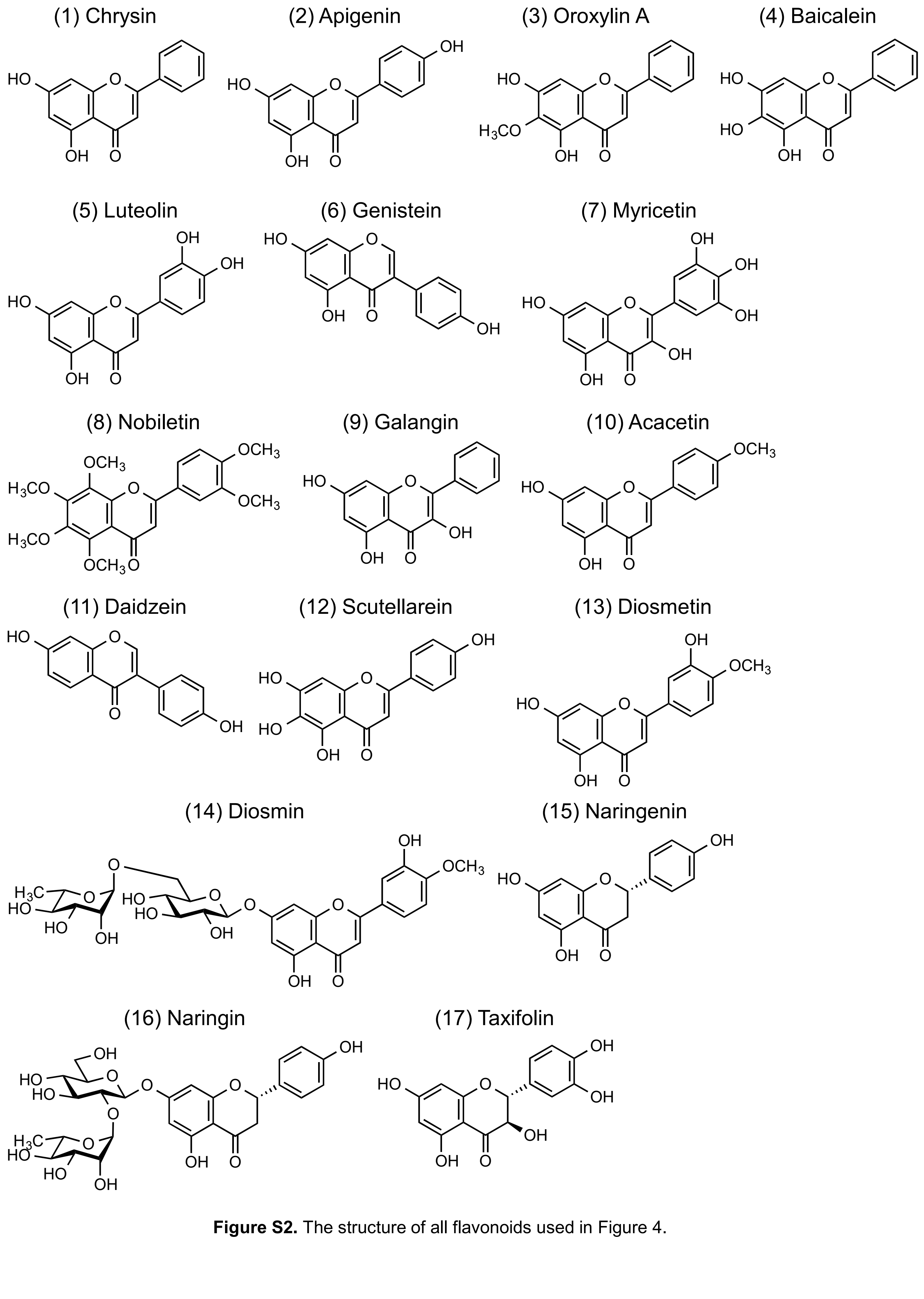

Figure S2. The structure of all flavonoids used in Figure 4.

(18) Hesperidin

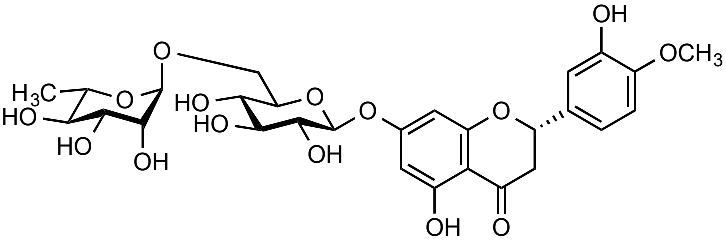

(19) Phloretin

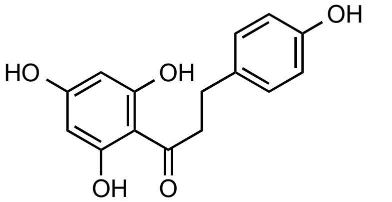

(20) Neohespridin dihydrochalcone

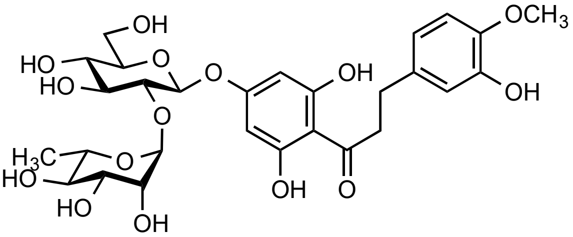

(21) Puerarin

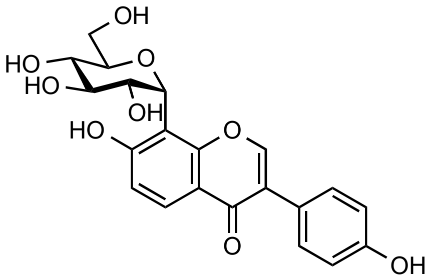

(22) Naringin dihydrochalcone

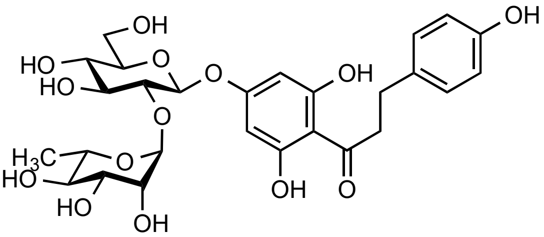

(23) Fisetin

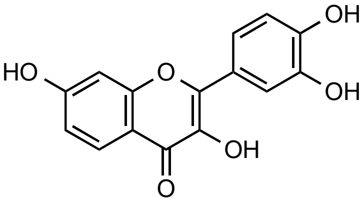

(24) Quercetin

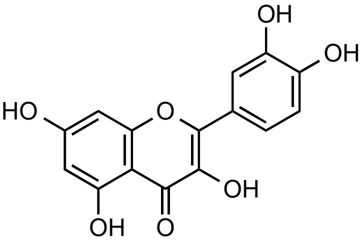

(25) Biochanin A

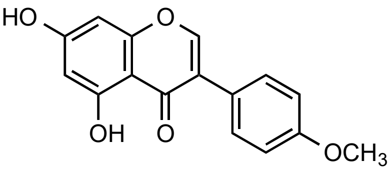

(26) Formononetin

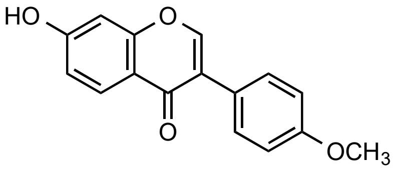

(27) Baicalin

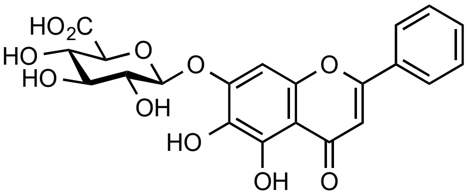

(28) Flavone

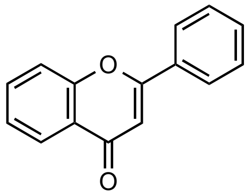

(29) 5-hydroxyflavone

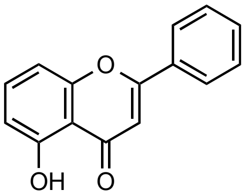

(30) 6-hydroxyflavone

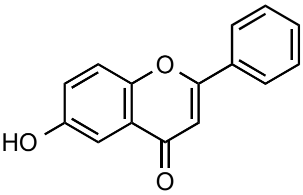

(31) 7-hydroxyflavone

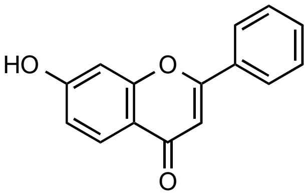

Figure S2. Cont.

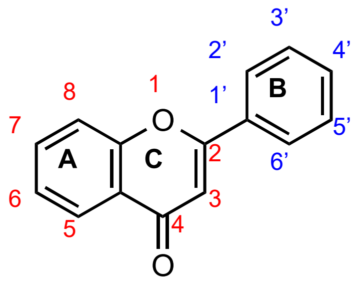

Figure S3. Chemical structure of flavone. Flavone has the structure consisting of fused A and C rings, and a phenyl B ring attached to position 2 of the C ring.
